# The TPx Protein of *Cysticercus cellulosae* Promotes an IL-10–Producing Regulatory B Cell Phenotype in Human B Lymphoblastoid Cells via the cAMP/CREB Pathway

**DOI:** 10.3390/cells15161429

**Published:** 2026-08-08

**Authors:** Haojun Cai, Xue Li, Haiting Xiong, Qianqian Mu, Biying Zhou

**Affiliations:** Department of Parasitology, School of Basic Medical Sciences, Zunyi Medical University, Zunyi 563000, China; 15672613277@163.com (H.C.); 15223811924@163.com (X.L.); xht15575261045@163.com (H.X.); 18061503055@163.com (Q.M.)

**Keywords:** *Taenia solium*, *Cysticercus cellulosae*, thioredoxin peroxidase, cAMP/CREB signaling pathway, Breg cell differentiatio

## Abstract

**Highlights:**

**What are the main findings?**

- The thioredoxin peroxidase (TPx) of *Cysticercus cellulosae* drives the human B lymphoblastoid line GM12878 to acquire an IL-10–producing CD19^+^CD24^hi^CD27^+^ regulatory B (Breg)-like phenotype.
- TPx induces Breg-like differentiation and IL-10 secretion through an adenylyl cyclase-dependent cAMP/CREB axis.

**What are the implications of the main findings?**

- TPx-conditioned B cells reduce CD4^+^ T-cell activation and increase a CD4^+^CD25^+^CD127^−^ Treg-like population in vitro, suggesting a Breg–Treg cooperative mechanism of immune evasion in cysticercosis.
- The cAMP/CREB axis represents a potential therapeutic target for cysticercosis and related parasitic diseases.

**Abstract:**

Thioredoxin peroxidase (TPx) derived from *Taenia solium* cysticerci (*Cysticercus cellulosae*) mediates immune evasion by disrupting T cell subset homeostasis. However, its effect on regulatory B cells (Bregs) and the underlying mechanism remain unknown. We stimulated GM12878 B cells with TPx and assessed Breg differentiation by flow cytometry, ELISA, and RT-qPCR. We evaluated the function of TPx-pretreated B cells in co-culture with T cells. We then combined RNA sequencing, Western blot, ELISA, and pharmacological inhibition to identify the signaling pathway involved. TPx increased the CD19^+^CD24^hi^CD27^+^ Breg subset in a time-dependent manner, peaking at 72 h. This effect was accompanied by enhanced IL-10 secretion and upregulated transcription of IL-10, TGF-β, and IL-35. TPx-pretreated B cells reduced CD4^+^ T-cell blastogenesis (cell size) and the CD4^+^ fraction of the co-culture and increased the frequency of cells with a CD4^+^CD25^+^CD127^−^ Treg-like surface phenotype. RNA sequencing identified cAMP-related pathways as central to this process. TPx elevated intracellular cAMP and activated the PKA/CREB phosphorylation cascade, whereas the adenylyl cyclase inhibitor SQ22536 reversed TPx-induced Breg differentiation and IL-10 secretion. In this human B cell line model, TPx promotes acquisition of a Breg-like phenotype in an adenylyl cyclase–dependent manner, revealing a novel mechanism of cysticercosis-associated immune evasion.

## 1. Introduction

Cysticercosis results from infection with the larval form of the pork tapeworm *Taenia solium* and remains an important zoonosis [1]. The World Health Organization includes it among the neglected tropical diseases requiring priority action [2]. When larvae establish in the central nervous system, neurocysticercosis (NCC) can develop; this condition is the most common infectious cause of acquired epilepsy globally and is associated with more than 50,000 deaths each year [3]. Current management relies on anthelmintic chemotherapy and surgery, both of which have substantial limitations: therapeutic drug concentrations are difficult to sustain in the brain, parasite death can trigger life-threatening secondary inflammation, and surgery carries inherent risks—collectively precluding a reliable cure [4]. Durable control may therefore benefit from vaccination, but rational vaccine design requires a clearer account of how cysticerci interact with and regulate host immunity.

Through prolonged co-evolution with their hosts, parasites have evolved sophisticated strategies to evade host immunity [5]. Central to these strategies are excretory–secretory antigens (ESAs)—bioactive molecules continuously released into the host microenvironment that modulate immune responses and sustain long-term parasite survival [6,7]. Thioredoxin peroxidase (TPx), an abundant ESA component in many helminths, exerts important immunomodulatory functions in infections caused by schistosomes, filarial nematodes, and other parasitic worms [8,9,10]. In *T. solium* cysticerci, TPx is widely expressed in the tegument and subtegumental muscle layers and is specifically recognized by sera from infected hosts, indicating strong immunogenicity [9,11,12]. Our earlier work showed that this protein shifted the Treg/Th17 balance by enhancing Treg cells and restraining Th17 differentiation through TGF-β/Smad signaling [13]. Most research on helminth TPx has nevertheless centered on macrophages or T-cell subsets [9,14], leaving its actions on B cells largely undefined.

Rather than constituting a single lineage, regulatory B (Breg) cells comprise pheno-typically diverse B cells defined by suppressive activity. Their effects can be mediated by IL-10, IL-35, TGF-β, or granzyme B, as well as by membrane molecules such as PD-L1 and FasL [15,16,17,18]. In parasitic infection, the induction of Breg expansion is increasingly recognized as a conserved evasion strategy that promotes chronicity: infections with *Schistosoma mansoni*, *Toxoplasma gondii*, *Leishmania* spp., and *Echinococcus* spp. all elicit large populations of IL-10–producing Breg cells that dampen protective T helper 1 (Th1) responses [19,20,21]. Notably, defined parasite-derived molecules—such as *S. mansoni* soluble egg antigen and *Echinococcus granulosus* excretory–secretory products—can directly drive Breg differentiation [22,23,24], suggesting that the exploitation of ESA components to induce Breg responses may be a broadly conserved parasitic strategy. Whether *T. solium* TPx likewise induces Breg differentiation, however, has not been reported.

IL-10 is a major functional output of Breg cells, although the signaling routes connecting extracellular cues to IL-10 transcription are not fully resolved. Studies in several immune-cell types point to the cyclic AMP (cAMP)–protein kinase A (PKA)–cAMP response element-binding protein (CREB) module [25,26,27]. Mechanistically, engagement of G protein-coupled receptors triggers Gαs-mediated activation of adenylyl cyclase and a consequent rise in intracellular cAMP; cAMP activates PKA, which phosphorylates CREB at serine 133 (Ser133) and promotes its nuclear translocation, thereby driving IL-10 transcription. Indeed, in Breg differentiation elicited by diverse stimuli—including CpG oligodeoxynucleotides, CD40 ligand, and mesenchymal stem cells—CREB Ser133 phosphorylation has been identified as a prerequisite for IL-10 transcriptional activation [27]. Whether this axis participates in Breg differentiation induced by parasite-derived molecules, however, has not been investigated.

Robust experimental models for dissecting these mechanisms are, however, constrained by the strict host specificity of *T. solium*, which naturally infects only humans and pigs. Clinical samples are difficult to obtain and display marked interindividual heterogeneity, while porcine models—despite recapitulating aspects of the host environment—are limited for molecular studies by their complex genetic background and the scarcity of species-specific reagents. We therefore employed two well-characterized human cell lines as complementary in vitro models: GM12878 is a karyotypically normal, non-malignant, EBV-immortalised human B lymphoblastoid line that has been characterised in exhaustive genomic, transcriptomic and epigenomic detail as an ENCODE Tier-1 reference line, and it offers high experimental reproducibility. EBV immortalisation nevertheless alters several immune-signalling and cytokine-regulatory networks. GM12878 was therefore used here as an exploratory system for identifying TPx-responsive B cell phenotypes and candidate signalling mechanisms, rather than as a substitute for primary human B cells [28,29,30,31]; and the Jurkat T lymphocyte line, an established system for studying T cell signaling, cytokine release, and receptor expression [32,33], which our group has previously used to define the role of TPx in Treg/Th17 balance [13].

On this basis, the present study aimed to (i) determine whether recombinant TPx induces Breg differentiation and IL-10 secretion; (ii) evaluate the effects of TPx-conditioned B cells on CD4^+^ T-cell blastogenesis (cell size), the CD4^+^ fraction of the co-culture, and the frequency of cells with a CD4^+^CD25^+^CD127^−^ Treg-like surface phenotype; and (iii) elucidate the role of the cAMP/CREB pathway in this process. We show that TPx promotes Breg-like differentiation and IL-10 production through an adenylyl cyclase-dependent cAMP/PKA/CREB axis, and that the resulting B cells reduce CD4^+^ T-cell blastogenesis and the CD4^+^ fraction of the co-culture while increasing the frequency of cells with a CD4^+^CD25^+^CD127^−^ Treg-like surface phenotype—providing a mechanistic framework for Breg-mediated immune evasion in cysticercosis.

## 2. Materials and Methods

### 2.1. Eukaryotic Expression and Purification of T. solium Cysticercus TPx Protein

The TPx coding region identified in our previous bioinformatic study [9] was synthesized and inserted into pcDNA3.4 downstream of a C-terminal 6×His tag. Eco-RI/BamHI digestion and Sanger sequencing were used to confirm pcDNA3.4-TPx. HEK293F cells were transfected with the validated construct using polyethylenimine (PEI; Cat. C0541; Beyotime Biotechnology, Shanghai, China). On day 6 after transfection, culture medium was collected and passed through Ni-NTA af-finity resin; bound TPx was recovered across a 20–200 mM imidazole gradient. SDS-PAGE (Figure S1) and Western blotting verified the expected molecular mass and purity [34]. An endotoxin-removal kit (Cat. C0268M; Beyotime Biotechnology) was then applied, and a separate detection kit (Cat. C0271S; Beyotime Biotechnology) confirmed a residual concentration below 0.01 EU/mL. Protein amount was measured by bicinchoninic acid assay (Cat. A045-4-2; Nanjing Jiancheng Bioengineering Institute, Nanjing, China), after which aliquots were frozen at −80 °C.

### 2.2. Cell Culture, Activation, and Co-Culture System

GM12878 B-lymphocyte cell line (Catalog No. SCSP-5244) and the human acute T-cell leukemia Jurkat cell line, Clone E6-1 (Catalog No. SCSP-513) were supplied by the Cell Bank of the Chinese Academy of Sciences (Shanghai, China). GM12878 cultures used RPMI 1640 (Cat. C11875500BT; Gibco, Grand Island, NY, USA) containing 15% fetal bovine serum (FBS; Cat. abs972; Absin, Shanghai, China), whereas Jurkat cultures contained 10% FBS. Both media included penicillin (100 U/mL) and streptomycin (100 μg/mL; Cat. P1400; Solarbio, Beijing, China). Cells were maintained at 37 °C under humidified 5% CO_2_ and were subcultured at approximately 80% confluence.

Experimental cultures contained 1 × 10^6^ cells in 2 mL per well of a 6-well plate. Before TPx exposure, every GM12878 experimental and control culture received 0.1 μM CpG ODN 2006 (Cat. HY-15021S; MedChemExpress, Monmouth Junction, NJ, USA) for 24 h to generate a standardized activation state [30]. This pre-treatment step was performed identically for all experimental and control groups. For TPx functional assays, TPx protein was then added to CpG-activated GM12878 cultures at a final concentration of 5 μg/mL, and cells and supernatants were harvested at 48 h and 72 h for downstream analyses. Jurkat cells were activated with 0.5 ng/mL PMA (Cat. P741; Solarbio, Beijing, China) plus 0.5 µM ionomycin (Cat. HY-126764; MedChemExpress, Monmouth Junction, NJ, USA) for 12 h, activated Jurkat cells were washed three times in PBS to remove residual PMA and ionomycin before co-culture.

For direct co-culture, TPx-exposed GM12878 cells were harvested and washed three times in phosphate-buffered saline (PBS), then combined with activated Jurkat cells for 72 h at a 1:1 ratio (1 × 10^6^ total cells per well in 6-well plates). Control B cells underwent the same CpG preactivation, washing, and culture procedures as TPx-primed B cells but were cultured without TPx before co-culture with activated Jurkat cells. To assess the involvement of the cAMP/CREB signaling pathway, the adenylyl cyclase inhibitor SQ22536 (Cat. HY-100396; MedChemExpress, Monmouth Junction, NJ, USA) was added at a final concentration of 100 μM and pre-incubated for 24 h before TPx treatment in the designated experimental groups.

### 2.3. Enzyme-Linked Immunosorbent Assay

Culture supernatant IL-10 was quantified with a human sandwich ELISA (Cat. EH0009; HUABIO, Hangzhou, China). Samples obtained 48 and 72 h after TPx addition were clarified at 1000× *g* for 10 min at 4 °C before analysis. For intracellular cAMP, cells were harvested, lysed as directed by the supplier, and analyzed with a competitive assay (Cat. BP02612; BPE, Shanghai, China). A SpectraMax iD3 reader (Molecular Devices, San Jose, CA, USA) recorded absorbance at 450 nm, and four-parameter logistic curves were used to derive concentrations. Each sample was tested in duplicate in three independent experiments.

### 2.4. RNA Extraction and Reverse Transcription–Quantitative PCR (RT-qPCR)

Total RNA was isolated from each group with TRIzol (Cat. R1100; Solarbio, Beijing, China). An Epoch microvolume spectrophotometer (BioTek Instruments, Winooski, VT, USA) was used to determine concentration and verify A260/A280 values of 1.8–2.0. For each sample, 1 μg RNA was converted to cDNA on a C1000 Touch cycler (Bio-Rad, Hercules, CA, USA) with PrimeScript RT reagent (Cat. RR037A; Takara, Kusatsu, Japan). Quantitative PCR used TB Green Premix Ex Taq II (Cat. RR820A; Takara) and a CFX96 system (Bio-Rad). The 20 μL mixture comprised 10 μL 2× premix, 2 μL cDNA, 1 μL of each 10 μM primer, and 6 μL nuclease-free water. Cycling began at 95 °C for 5 min and contin-ued for 40 cycles of 95 °C for 30 s, 58 °C for 30 s, and 72 °C for 30 s. Melt-curve analysis and a no-template control were included in each run. Table S1 lists the primers. GAPDH served as the reference for relative quantification by the 2^−ΔΔCt^ calculation. Three independent experiments were performed.

### 2.5. Flow Cytometry

After collection and PBS washing, cells were prepared as single-cell suspensions. For extracellular labeling, 1 × 10^6^ cells were placed in each tube and incubated for 30 min at 4 °C, protected from light, with the indicated fluorochrome-conjugated antibodies. All antibodies used for cytometry were obtained from BD Pharmingen (BD Biosciences, San Die-go, CA, USA).

B-cell staining panel. GM12878 cells were stained with anti-CD19-FITC (clone HIB19), anti-CD24-PE-Cy7 (clone ML5), and anti-CD27-APC (clone M-T271). For analysis of activation during CpG optimization, GM12878 cells were stained with anti-CD19-FITC and anti-CD69-PE (clone FN50). Following exclusion of debris and doublets, the CD19^+^ population was identified, and CD24^hi^CD27^+^ cells were quantified within the CD19^+^ parent gate. The CD24^hi^ gate was defined using the same fluorescence threshold across all treatment groups within each independent experiment.

Intracellular IL-10 panel. cells were restimulated for 5 h at 37 °C with 50 ng/mL PMA, 1 µg/mL ionomycin and 10 µg/mL brefeldin A (Cat. abs810012; Absin, Shanghai, China); surface staining was performed as above, followed by fixation/permeabilisation with the BD Cytofix/Cytoperm kit (Cat. 554722; BD Biosciences). IL-10^+^ cells were quantified within the sequentially gated CD19^+^CD24^hi^CD27^+^ population. In the TPx dose-optimization experiment, CD19^+^IL-10^+^ cells were additionally quantified within the CD19^+^ gate.

T-cell staining panel. Jurkat cells from the T-cell-only and B–T-cell co-culture experiments were stained with anti-CD4-APC (clone RPA-T4), anti-CD25-PE (clone M-A251), and anti-CD127-FITC (clone HIL-7R-M21). CD25^+^CD127^−^ cells were quantified within the CD4^+^ parent gate.

At least 50,000 lymphocyte-gated events per sample were collected on a Cytek North-ern Lights spectral cytometer (Cytek Biosciences, Fremont, CA, USA). An individual single-stained reference control was acquired for each fluorochrome and used to establish the corresponding spectral reference signature. The resulting unmixing matrix was applied consistently to all samples within each experiment. The unmixed FCS files were subsequently analyzed using FlowJo v10 (BD Life Sciences, Ashland, OR, USA) with an identical gating strategy across experimental groups.

### 2.6. RNA Sequencing (RNA-Seq) and Bioinformatics Analysis

Three biological replicates of TPx-treated GM12878 cells and three matched untreated controls were harvested for RNA isolation with TRIzol. RNA quality was evaluated from the A260/A280 ratio and RNA integrity number (RIN), and only samples with RIN ≥ 7.0 proceeded to analysis. Shanghai Applied Protein Technology Co., Ltd. (Shanghai, China) prepared strand-specific libraries and generated paired-end reads on an Illumina No-vaSeq 6000 (Illumina, Inc., San Diego, CA, USA). After quality filtering, reads were mapped to GRCh38 with HISAT2 and summarized at gene level by featureCounts (version 2.1.1, Subread package). Differential expression required *p* < 0.05 together with |log_2_ fold change| > 0.58, equivalent to a change greater than 1.5-fold [35].

DAVID v6.8 (https://davidbioinformatics.nih.gov/, accessed on 6 May 2025) was used to annotate the differentially ex-pressed genes and test functional enrichment. Gene Ontology results were grouped into biological process, cellular component, and molecular function, and pathway annotations were drawn from KEGG. Enrichment was evaluated by Fisher’s exact test. Benjamini–Hochberg adjustment addressed multiple testing, with adjusted *p* < 0.05 defining significance.

### 2.7. Western Blot Analysis

Cells were incubated on ice for 30 min in RIPA buffer (Cat. R0010; Solarbio, Beijing, China) containing phosphatase inhibitors (Cat. P1260; Solarbio). After centrifugation at 8000× *g* for 15 min at 4 °C, supernatant protein was measured by the BCA procedure de-scribed above. Ten micrograms per lane were resolved on 10% SDS-PAGE and transferred to 0.45 μm PVDF at 400 mA for 30 min. Membranes were blocked for 1 h at room temperature in 5% non-fat milk prepared in TBST and incubated at 4 °C overnight with phospho-CREB Ser133 (1:2000; Cat. 28792-1-AP), CREB (1:3000; Cat. 12208-1-AP), PKA catalytic subunit α (1:3000; Cat. 27398-1-AP), or GAPDH (1:5000; Cat. 81640-5-RR); all primary antibodies were from Proteintech (Wuhan, China). Following three TBST washes, HRP-linked secondary antibody (Cat. abs20040; Absin, Shanghai, China) was applied for 1 h at room temperature. Signals were developed with ECL reagent (Cat. MA0186-1; Mei-lunBio, Dalian, China), captured using a ChemiDoc MP (Bio-Rad), and quantified in Image Lab 6.1 relative to GAPDH. Immunoblots were repeated independently at least three times.

### 2.8. CCK-8 Cell Viability Assay

A CCK-8 assay was used to select an SQ22536 concentration that did not cause overt short-term loss of viability. Log-phase GM12878 cells were dispensed into 96-well plates at 2 × 10^4^ cells in 100 μL per well and exposed to 50, 100, 200, or 400 μM SQ22536. Each condition had five wells; matched DMSO and medium-only wells served as vehicle and blank controls. Following 24 h at 37 °C and 5% CO_2_, 10 μL CCK-8 reagent (Cat. B6005M; BMC Life Science, Beijing, China) was added. Absorbance at 450 nm was recorded after a further 3 h, and viability was computed as (OD_treatment_ − OD_blank_)/(OD_control_ − OD_blank_) × 100%. The experiment was independently repeated three times.

### 2.9. Statistical Analysis

Values are reported as mean ± SD from at least three independent experiments. Distributional normality and equality of variance were examined with Shapiro–Wilk and Levene tests, respectively. When parametric assumptions were met, two groups were compared by a two-sided independent-samples Student’s *t*-test, and multiple groups by one-way ANOVA with Tukey HSD comparisons. Otherwise, Mann–Whitney U or Krus-kal–Wallis tests were used for two- or multi-group analyses. Calculations were performed in SPSS 29.0 (IBM Corp., Armonk, NY, USA), and GraphPad Prism 9.0 (GraphPad Soft-ware, San Diego, CA, USA) was used for plotting. Statistical significance was defined by a two-sided *p* < 0.05.

## 3. Results

### 3.1. TPx Protein Induces Differentiation of GM12878 B Cells into IL-10-Producing Breg Cells

**Figure 1:**
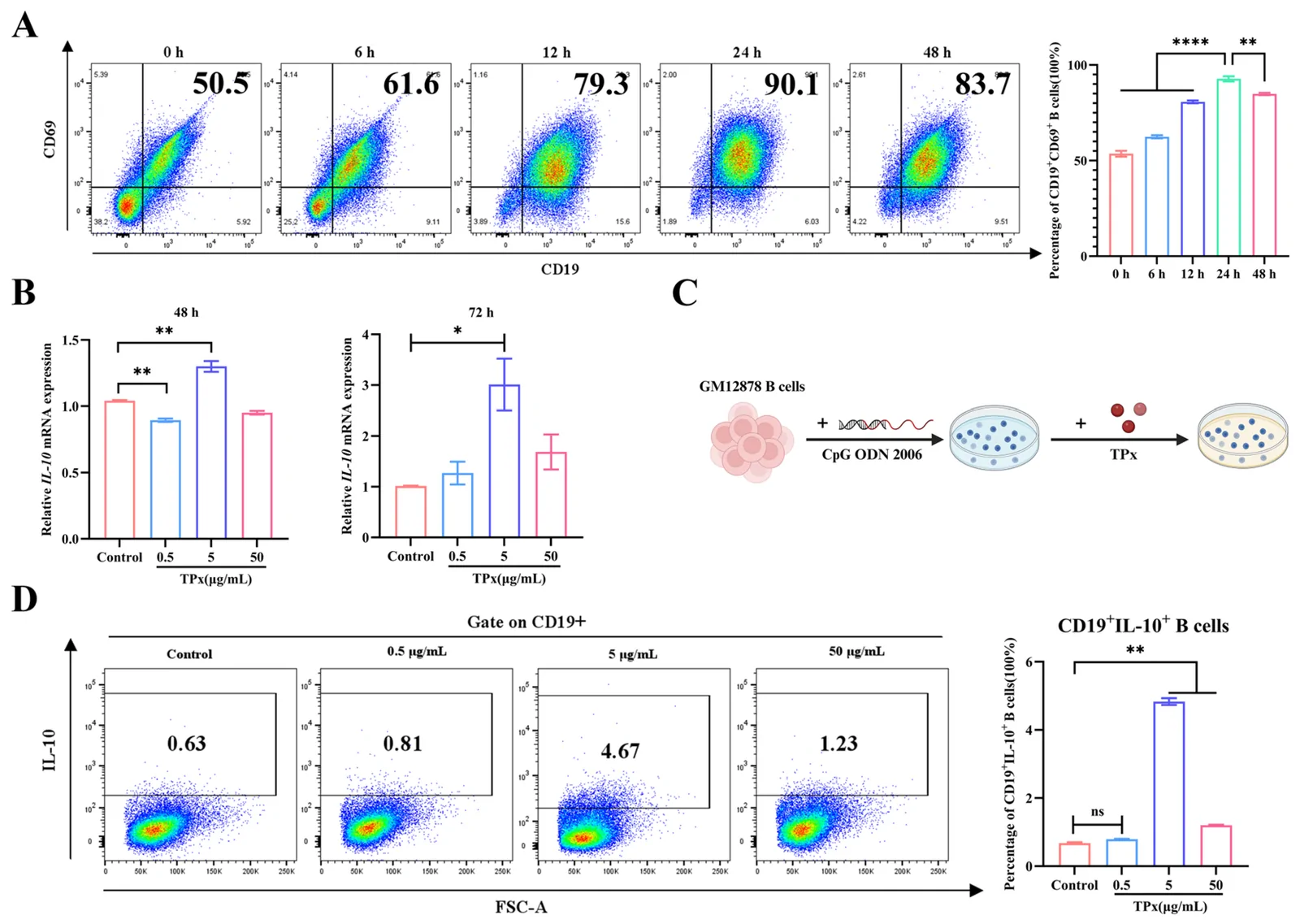
Optimization of CpG ODN 2006 activation time and TPx concentration for inducing IL-10 expression in GM12878 B cells. (**A**)—Representative flow cytometry dot plots and quantification of activated CD19^+^CD69^+^ B cells at 0, 6, 12, 24, and 48 h after CpG ODN 2006 stimulation. The proportion of CD19^+^CD69^+^ cells increased progressively, peaking at 24 h (90.1%). (**B**)—Relative IL-10 mRNA expression in CpG-preactivated GM12878 B cells treated with TPx (0.5, 5, or 50 μg/mL) for 48 h (left) and 72 h (right), determined by qPCR; expression peaked at 5 μg/mL at both time points. (**C**)—Schematic of the experimental workflow: GM12878 B cells were preactivated with CpG ODN 2006 for 24 h and then stimulated with TPx at the indicated concentrations. (**D**)—Representative flow cytometry dot plots (gated on CD19^+^ cells) and quantification of CD19^+^IL-10^+^ B cells after treatment with the indicated TPx concentrations; the frequency of IL-10-producing B cells was maximal at 5 μg/mL (4.67%). Together, these data identify 24 h of CpG ODN 2006 preactivation and 5 μg/mL TPx as the optimal conditions for inducing IL-10 expression. Data are presented as mean ± SD. ns, not significant; * *p* < 0.05; ** *p* < 0.01; **** *p* < 0.0001.

CpG ODN 2006 stimulation progressively activated GM12878 B cells, with CD19^+^CD69^+^ frequencies rising over time and exceeding 90% by 24 h (*p* < 0.0001; Figure 1A). Accordingly, 24 h of CpG pre-activation was adopted for all subsequent experiments (Figure 1C). Among the TPx doses tested, 5 μg/mL most strongly induced IL-10 expression: IL-10 mRNA peaked in the 5 μg/mL group at both 48 h and 72 h (Figure 1B), and CD19^+^IL-10^+^ cells reached 4.67% at this dose versus ≤1.23% in all other groups (*p* < 0.01; Figure 1D). The response was non-monotonic across the three concentrations tested—0.5 μg/mL was ineffective (*p* > 0.05), and 50 μg/mL was markedly less potent than 5 μg/mL—identifying 5 μg/mL as the most effective concentration under our conditions. The basis of the reduced high-dose response remains unresolved: viability, apoptosis, protein aggregation, and negative-feedback signalling were not separately assessed in the 50 μg/mL group, so possibilities such as receptor desensitization, competing inhibitory signalling, or dose-dependent cytotoxicity remain to be tested directly. On this basis, 5 μg/mL for 72 h was adopted as the standard treatment condition.

**Figure 2:**
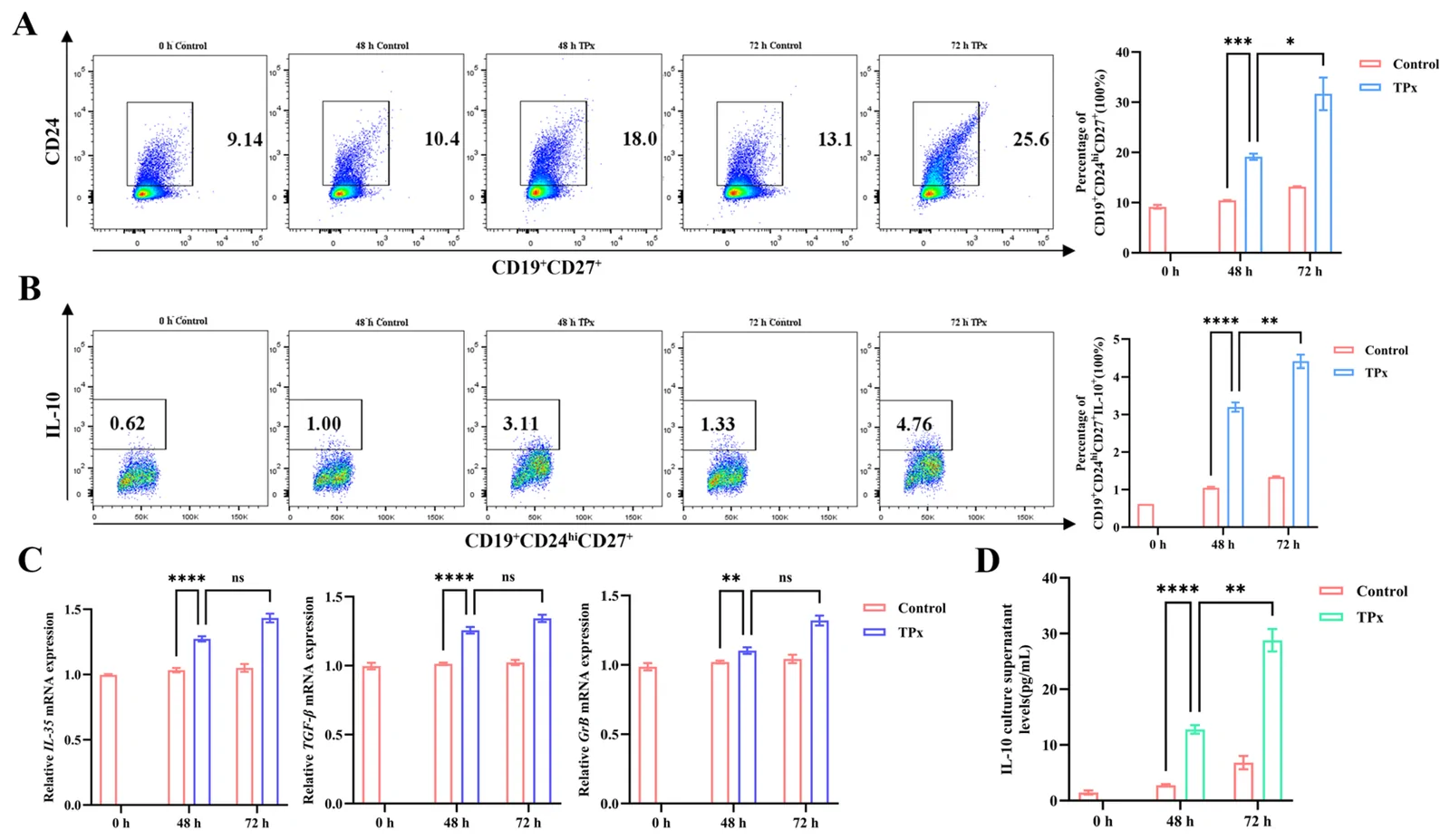
TPx promotes differentiation of GM12878 B cells into CD19^+^CD24^hi^CD27^+^ IL-10-producing Breg cells. (**A**)—Representative flow cytometry dot plots (gated on CD19^+^ cells) and quantification of CD19^+^CD24^hi^CD27^+^ Breg cells at 0, 48, and 72 h in control and TPx-treated (5 μg/mL) groups. TPx significantly increased the Breg proportion over time, reaching 25.6% at 72 h versus 13.1% in the time-matched control. (**B**)—Representative flow cytometry dot plots and quantification of IL-10^+^ cells within the CD19^+^CD24^hi^CD27^+^ gated population at the indicated time points; TPx markedly elevated the frequency of IL-10-producing Breg cells, peaking at 4.76% at 72 h compared with 1.33% in the control. (**C**)—Relative mRNA expression of the Breg-associated effector molecules IL-35, TGF-β, and GZMB in control and TPx-treated groups at 0, 48, and 72 h, determined by qPCR; TPx significantly upregulated IL-35, TGF-β, and GZMB expression at 48 h, though the differences were not statistically significant at 72 h (ns). (**D**)—IL-10 protein concentrations in culture supernatants of control and TPx-treated groups at 0, 48, and 72 h, measured by ELISA; TPx significantly enhanced IL-10 secretion in a time-dependent manner, reaching ~29 pg/mL at 72 h. Together, these data demonstrate that TPx drives the differentiation of GM12878 B cells into IL-10-producing Breg cells and enhances expression of their key immunoregulatory effector molecules. Data are presented as mean ± SD. ns, not significant; * *p* < 0.05; ** *p* < 0.01; *** *p* < 0.001; **** *p* < 0.0001.

Under these conditions, TPx drove a time-dependent expansion of CD19^+^CD24^hi^CD27^+^ Breg cells (from 18.0% at 48 h to 25.6% at 72 h vs. ≤13.1% in time-matched controls; *p* < 0.05; Figure 2A), a parallel increase in IL-10^+^ cells within this subset (from 3.11% to 4.76%; *p* < 0.01; Figure 2B), and a corresponding elevation in secreted IL-10, which reached ~29 pg/mL at 72 h (*p* < 0.01; Figure 2D). Analysis of additional Breg effector molecules revealed that IL-35, TGF-β, and GZMB were all significantly upregulated at 48 h but plateaued thereafter, showing no further significant change at 72 h (Figure 2C). In contrast, IL-10—at both the transcriptional and protein levels—continued to rise through 72 h, identifying it as the principal and most dynamically regulated effector of TPx-induced Bregs.

Collectively, these results demonstrate that TPx promotes the differentiation of GM12878 B cells into CD19^+^CD24^hi^CD27^+^ Breg cells in a dose- and time-dependent manner, with IL-10 as the dominant functional output.

### 3.2. TPx-Induced Breg Cells Suppress CD4^+^ T Cell Immune Responses

**Figure 3:**
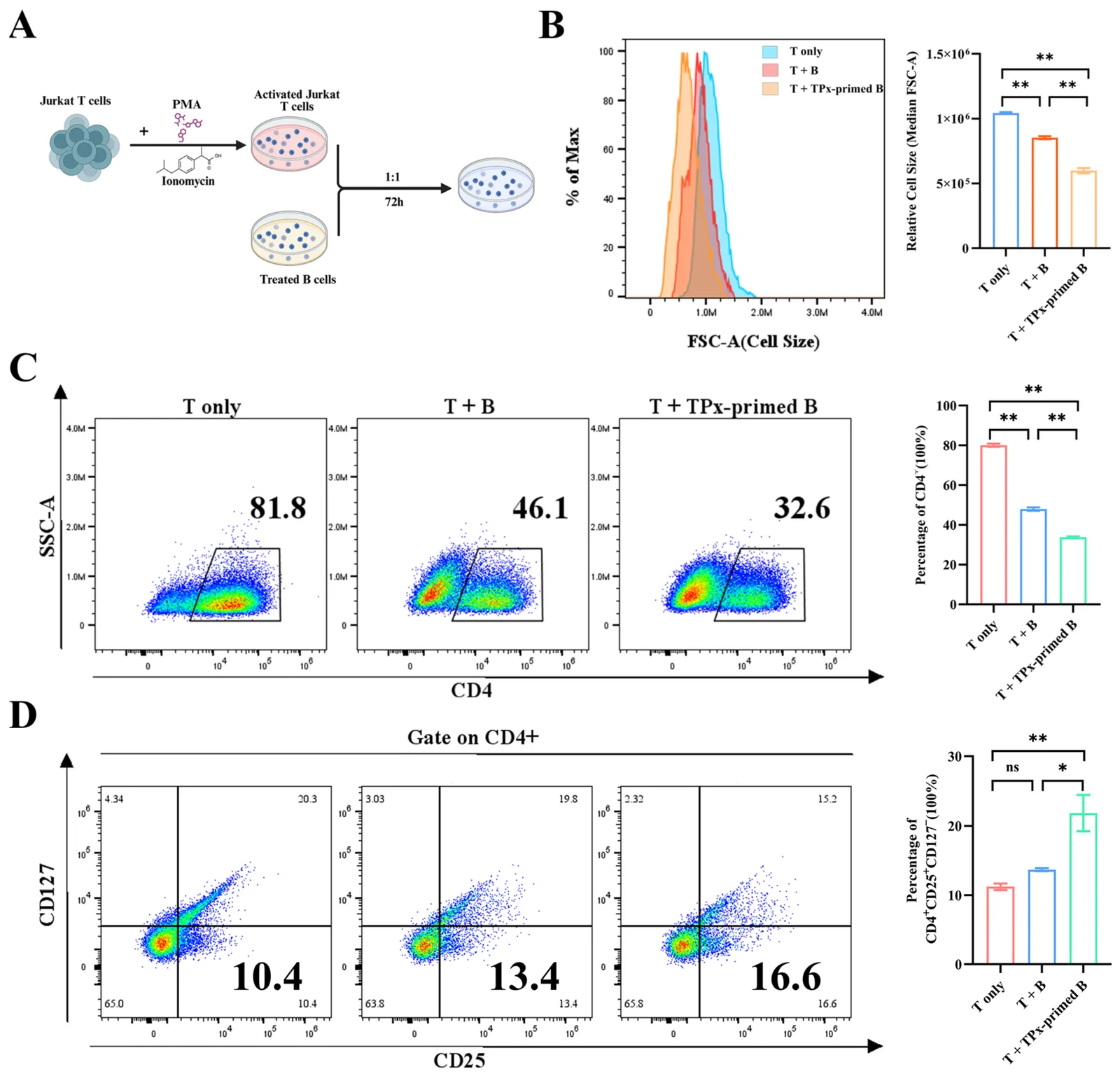
TPx-primed B cells reduce CD4^+^ Jurkat T-cell blastogenesis and increase the frequency of a CD4^+^CD25^+^CD127^−^ Treg-like phenotype in a B–T co-culture system. (**A**)—Schematic of the co-culture workflow: Jurkat T cells were activated with PMA plus ionomycin and co-cultured at a 1:1 ratio with either untreated B cells (T + B) or TPx-primed B cells (T + TPx-primed B) for 72 h; activated T cells cultured alone (T only) served as the reference. (**B**)—Representative FSC-A histograms (left) and quantification of median FSC-A values (right) of Jurkat T cells across the three groups, reflecting relative cell size as an indicator of T cell blastogenesis. T cell size decreased progressively, with TPx-primed B cells producing the greatest reduction. (**C**)—Representative flow cytometry dot plots and quantification of CD4^+^ T cell proportions across the three groups. The CD4^+^ proportion declined from 81.8% (T only) to 46.1% (T + B) and further to 32.6% (T + TPx-primed B), indicating enhanced suppression of CD4^+^ T cell activation by TPx-primed B cells. (**D**)—Representative flow cytometry dot plots (gated on CD4^+^ cells) and quantification of CD4^+^CD25^+^CD127^−^ Treg cell proportions across the three groups; TPx-primed B cells significantly increased the Treg proportion (16.6%) relative to untreated B cells (13.4%) and T only (10.4%). Together, these data demonstrate that TPx-induced Breg cells suppress CD4^+^ T-cell blastogenesis (cell size) and reduce the CD4^+^ fraction in the co-culture, while increasing the frequency of cells with a CD4^+^CD25^+^CD127^−^ Treg-like surface phenotype. Data are presented as mean ± SD. ns, not significant; * *p* < 0.05; ** *p* < 0.01.

To assess the immunosuppressive function of TPx-induced Breg cells, PMA/ionomycin-activated Jurkat T cells were co-cultured (1:1, 72 h) with either untreated or TPx-induced B cells, and T cell activation and differentiation were analyzed by flow cytometry (Figure 3A).

T cell blastogenesis, measured by forward scatter (FSC-A), was progressively reduced across the three conditions: activated T cells alone showed the largest, most proliferative phenotype, co-culture with untreated B cells lowered FSC-A, and co-culture with TPx-induced Breg cells produced the smallest, most quiescent cells (all pairwise *p* < 0.01; Figure 3B). CD4^+^ T cell frequencies followed the same hierarchy, falling from 81.8% (T alone) to 46.1% (untreated B) and 32.6% (TPx-induced Breg) (all pairwise *p* < 0.01; Figure 3C). Thus, TPx-induced Breg cells suppress CD4^+^ T cell blastogenesis and expansion more potently than untreated B cells.

Analysis of the CD4^+^ population showed that the frequency of cells with a CD4^+^CD25^+^CD127^−^ Treg-like surface phenotype was significantly increased only in co-cultures with TPx-conditioned B cells (16.6% vs. 13.4% for untreated B, *p* < 0.05), whereas untreated B cells did not differ significantly from T cells alone (*p* > 0.05; Figure 3D). This indicates that the increase in cells with a CD4^+^CD25^+^CD127^−^ Treg-like surface phenotype is actively driven by TPx-induced Breg cells, rather than simply reflecting generalized T-cell activation.

Together, these results indicate that TPx-conditioned B cells exert bidirectional immunoregulation—reducing CD4^+^ T-cell blastogenesis (cell size) and the CD4^+^ fraction of the co-culture, while increasing the frequency of cells with a CD4^+^CD25^+^CD127^−^ Treg-like surface phenotype.

### 3.3. Transcriptomic Analysis Implicates the cAMP Signaling Pathway in TPx-Induced B Cell Reprogramming

**Figure 4:**
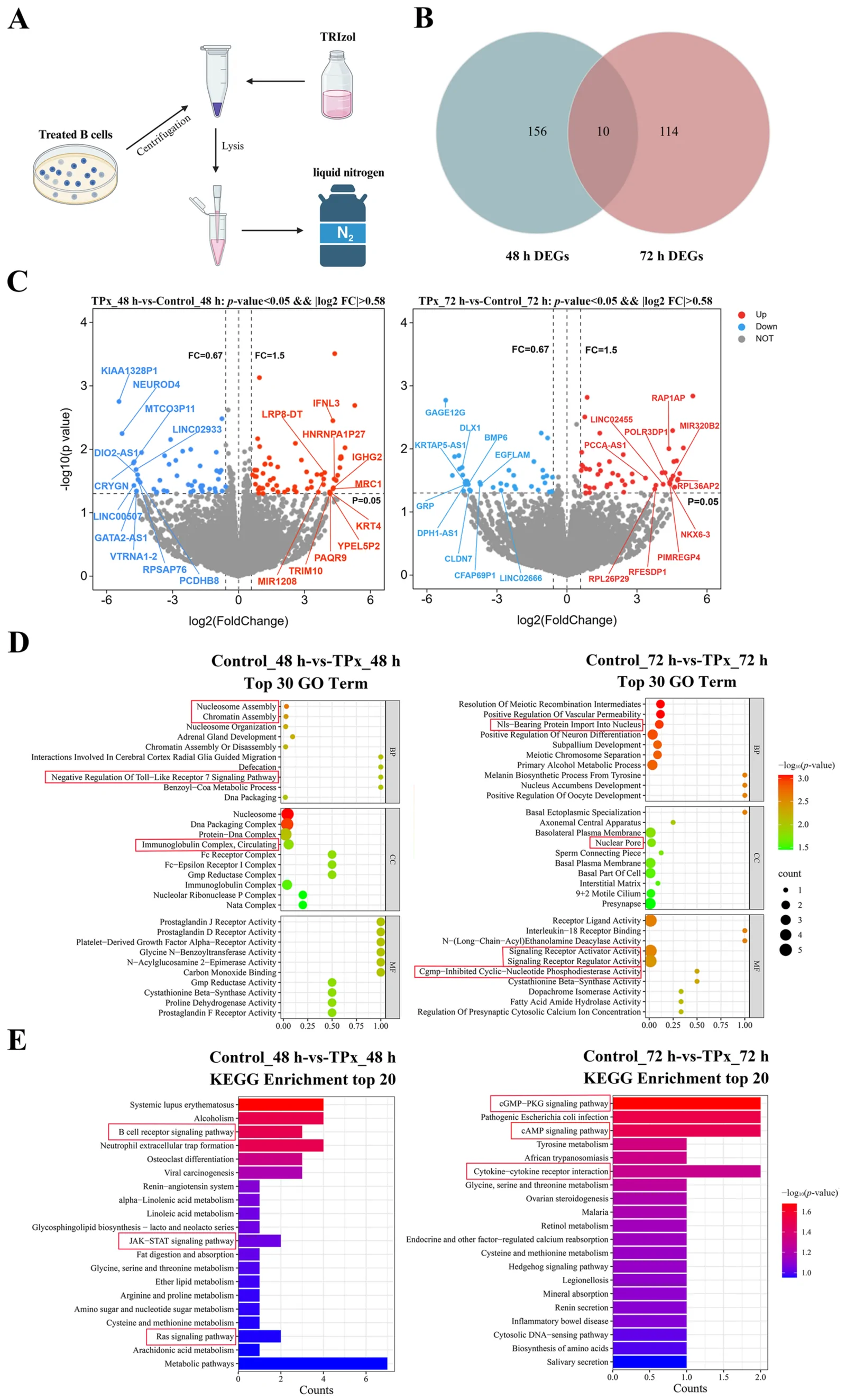
Transcriptomic profiling of TPx-treated GM12878 B cells reveals time-dependent transcriptional reprogramming and enrichment of cyclic nucleotide signaling pathways. (**A**)—Schematic of the RNA extraction and sample preparation workflow for transcriptomic sequencing. Total RNA was extracted from TPx-treated and time-matched control B cells using the TRIzol method, and samples were snap-frozen in liquid nitrogen prior to sequencing. (**B**)—Venn diagram showing the overlap of differentially expressed genes (DEGs) identified at 48 h and 72 h of TPx treatment; 156 DEGs were unique to 48 h, 114 unique to 72 h, and 10 shared between the two time points. (**C**)—Volcano plots of DEGs at 48 h (left) and 72 h (right) of TPx treatment relative to time-matched controls. Red, blue, and gray dots represent significantly upregulated, significantly downregulated, and non-significant genes, respectively (thresholds: *p* < 0.05 and |log_2_ fold change| > 0.58). Representative gene names are labeled. (**D**)—Gene Ontology (GO) enrichment analysis of DEGs at 48 h (left) and 72 h (right), showing the top 30 enriched terms across biological process (BP), cellular component (CC), and molecular function (MF) categories. Dot size indicates gene count, and color intensity represents −log_10_ (*p* value). Terms highlighted with red boxes denote immune- and signaling-related processes, including negative regulation of Toll-like receptor 7 signaling and cGMP-inhibited cyclic-nucleotide phosphodiesterase activity. (**E**)—KEGG pathway enrichment analysis of DEGs at 48 h (left) and 72 h (right), displaying the top 20 enriched pathways. Bar length indicates gene count, and color intensity represents −log_10_ (*p* value). Pathways highlighted with red boxes denote key immunoregulatory and signaling pathways, including B cell receptor, JAK-STAT, and Ras signaling at 48 h and cGMP-PKG, cAMP, and cytokine–cytokine receptor interaction pathways at 72 h.

To define the molecular basis of TPx-induced immunoregulation, RNA sequencing was performed on TPx-treated and time-matched control B cells (Figure 4A). TPx treatment yielded 166 differentially expressed genes (DEGs; 86 up, 80 down) at 48 h and 124 DEGs (69 up, 55 down) at 72 h (Figure 4C; Table S2). Only 10 DEGs were shared between the two time points (~3.6% overlap; Figure 4B), indicating that TPx engages largely distinct, stage-specific transcriptional programs.

GO enrichment (Figure 4D) reflected this temporal shift. At 48 h, DEGs were dominated by chromatin/nucleosome assembly (BP, CC) together with immune terms such as immunoglobulin complex, circulating and negative regulation of Toll-like receptor 7 signaling; notably, the genes driving the latter term were upregulated by TPx, a pattern compatible with—but not demonstrative of—dampened TLR7 signaling. These enrichments are hypothesis-generating and, in the absence of protein-level or functional validation, should be interpreted as nominating early effects on the epigenetic landscape and immunoglobulin-related functions rather than establishing them. By 72 h, enrichment shifted toward cGMP-inhibited cyclic-nucleotide phosphodiesterase activity (MF), nuclear pore (CC), and NLS-bearing protein import into nucleus (BP), pointing to later regulation of second-messenger metabolism and nucleocytoplasmic transport.

KEGG analysis (Figure 4E) showed a parallel progression. Early (48 h) DEGs were annotated to immunoregulatory pathways—systemic lupus erythematosus, B cell receptor, JAK–STAT, and Ras signaling—broadly consistent with the humoral/immunoglobulin GO terms, although pathway membership alone does not indicate whether these programs were activated or repressed. Late (72 h) DEGs converged on cyclic-nucleotide second-messenger signaling (cGMP–PKG and cAMP pathways) and cytokine–cytokine receptor interaction.

Collectively, these data suggest that TPx reprograms B cells in two phases—an early epigenetic/humoral program followed by a later shift toward cyclic-nucleotide (notably cAMP) signaling—nominating the cAMP pathway as a candidate mediator of TPx-induced Breg function.

### 3.4. TPx Promotes Breg-like Differentiation and IL-10 Production Through an Adenylyl Cyclase-Dependent cAMP/CREB Pathway

**Figure 5:**
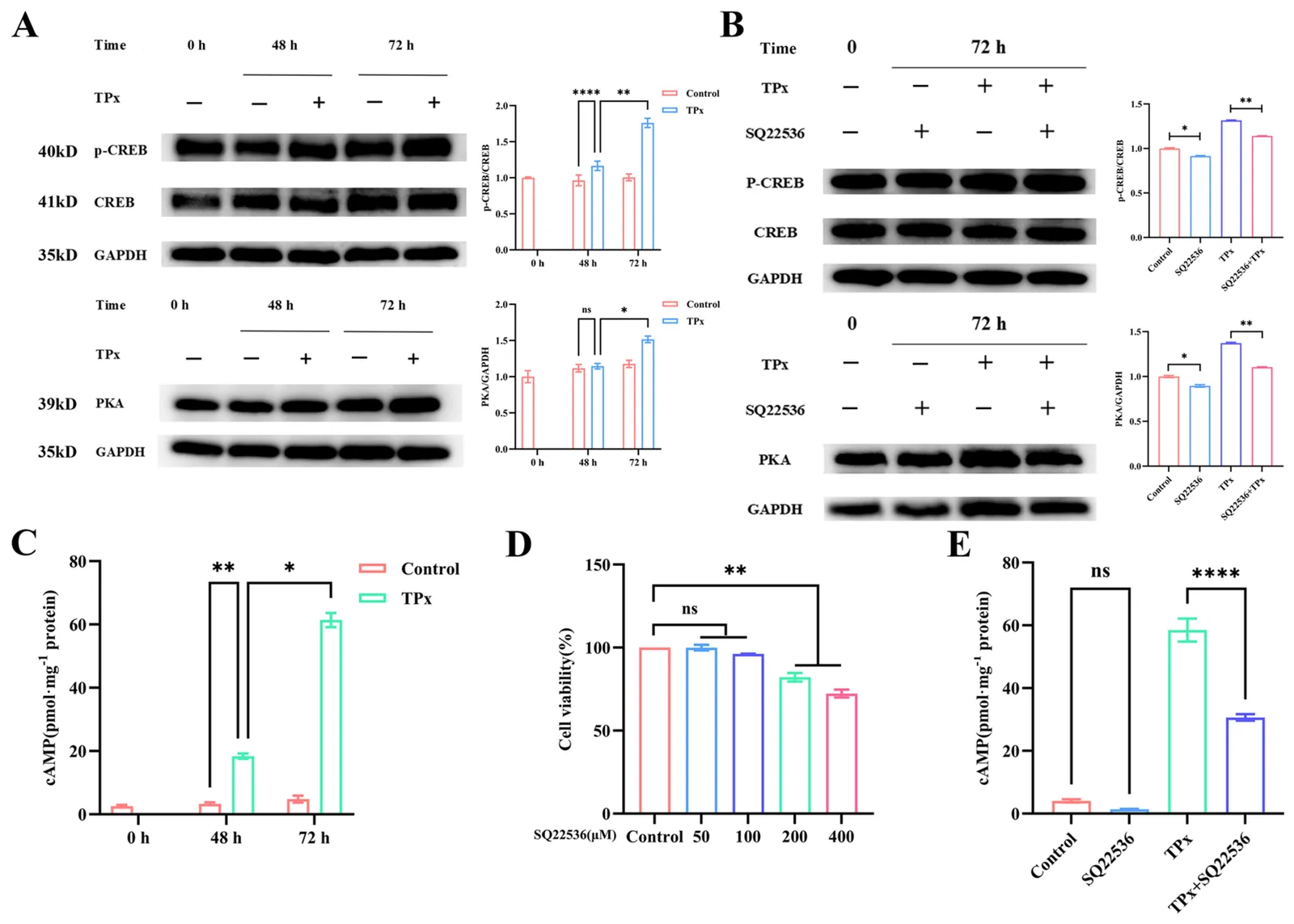
TPx activates the cAMP/PKA/CREB signaling cascade in GM12878 B cells in a time-dependent manner, and pharmacological inhibition of adenylyl cyclase attenuates pathway activation. (**A**)—Representative Western blot images and densitometric quantification of the p-CREB/CREB ratio (upper) and PKA/GAPDH ratio (lower) in control and TPx-treated B cells at 0, 48, and 72 h. GAPDH served as the loading control. TPx progressively increased both p-CREB and PKA levels, peaking at 72 h. (**B**)—Representative Western blot images and densitometric quantification of the p-CREB/CREB ratio (upper) and PKA/GAPDH ratio (lower) at 72 h across four treatment groups: Control, SQ22536 (100 μM), TPx (5 μg/mL), and SQ22536 + TPx. The adenylyl cyclase inhibitor SQ22536 significantly reduced TPx-induced p-CREB and PKA expression. (**C**)—Intracellular cAMP concentrations in control and TPx-treated B cells at 0, 48, and 72 h, measured by ELISA; TPx markedly elevated cAMP levels in a time-dependent manner, reaching ~61 pmol/mg protein at 72 h. (**D**)—Cell viability of GM12878 B cells treated with SQ22536 at 50, 100, 200, and 400 μM, assessed by CCK-8 assay; viability was unaffected at ≤100 μM but significantly decreased at ≥200 μM. (**E**)—Intracellular cAMP concentrations at 72 h across the four treatment groups (Control, SQ22536, TPx, and SQ22536 + TPx), measured by ELISA; SQ22536 significantly suppressed TPx-induced cAMP accumulation. Together, these data demonstrate that TPx activates the cAMP/PKA/CREB signaling cascade, which is attenuated by adenylyl cyclase inhibition. Data are presented as mean ± SD. ns, not significant; * *p* < 0.05; ** *p* < 0.01; **** *p* < 0.0001.

Pathway engagement was time-dependent (Figure 5A,C): at 48 h only the p-CREB/CREB ratio was significantly increased (*p* < 0.05), with total PKA Cα protein and whole-cell cAMP unchanged (*p* > 0.05), whereas at 72 h all three readouts were significantly elevated—p-CREB/CREB (*p* < 0.01), PKA (*p* < 0.05) and cAMP (*p* < 0.05). CREB Ser133 phosphorylation therefore preceded detectable whole-cell cAMP accumulation and PKA Cα induction. Two methodological caveats should be noted in interpreting this sequence: total PKA Cα protein reports pathway abundance rather than the allosteric activation state of the holoenzyme, and steady-state whole-cell cAMP measured at a single late time point is insensitive to transient or compartmentalised cAMP signals. The early increase in p-CREB may therefore reflect either a cAMP/PKA input not captured by these assays or the activity of cAMP-independent CREB Ser133 kinases. All subsequent pharmacological analyses were performed at 72 h, when the complete cascade and Breg differentiation were maximal.

To test pathway dependence, the adenylyl cyclase inhibitor SQ22536 was applied at 72 h. CCK-8 assays showed no toxicity at 50–100 μM but significant loss of viability at 200–400 μM (*p* < 0.01; Figure 5D), so 100 μM was used. SQ22536 alone lowered baseline p-CREB/CREB and PKA (*p* < 0.01 and *p* < 0.05; Figure 5B), and in combination it blunted the TPx-induced rise in p-CREB (*p* < 0.05) and PKA (*p* < 0.01). Consistently, SQ22536 did not alter basal cAMP (*p* > 0.05) but sharply reduced TPx-induced cAMP accumulation (*p* < 0.0001; Figure 5E).

**Figure 6:**
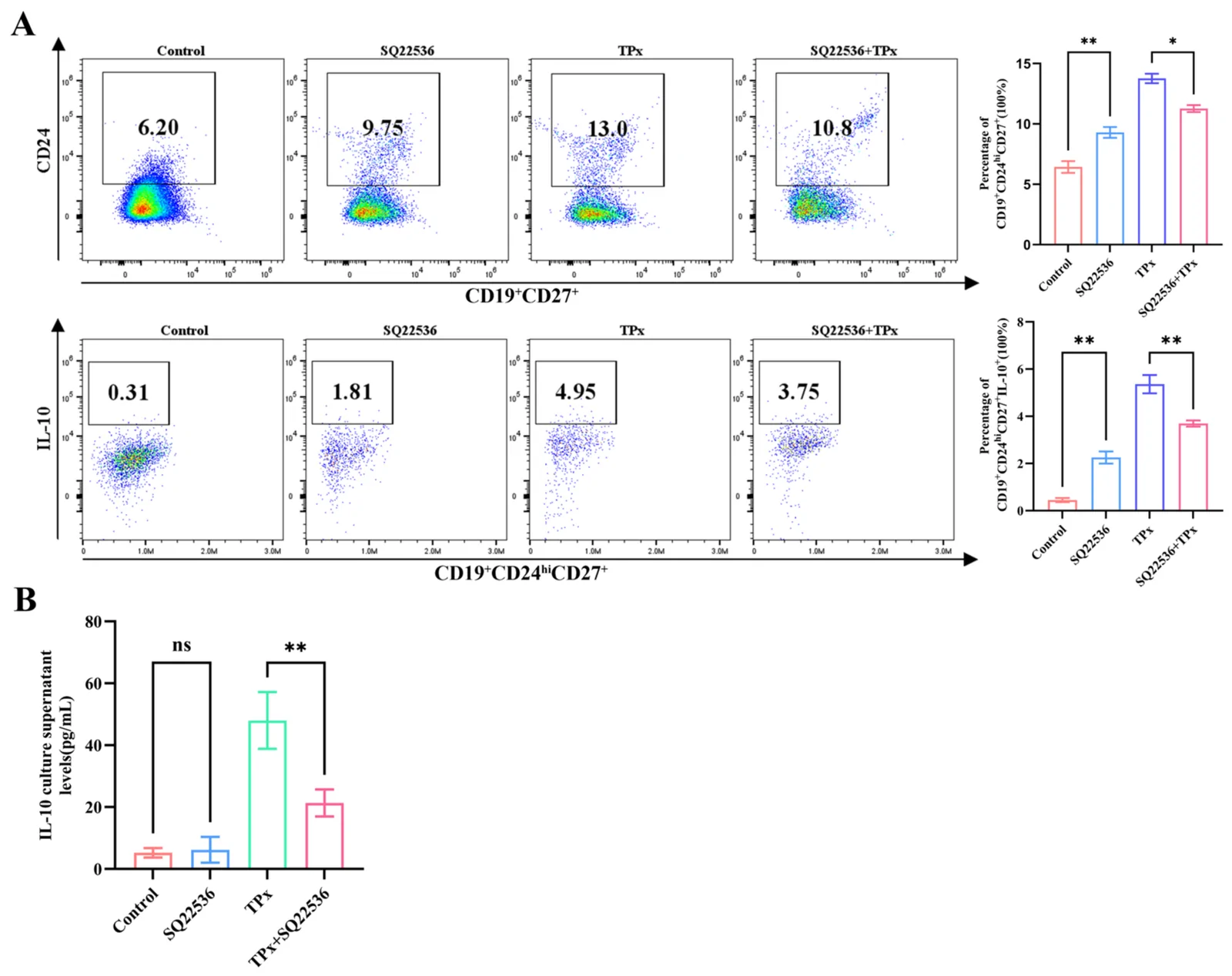
Pharmacological inhibition of adenylyl cyclase attenuates TPx-induced Breg cell differentiation and IL-10 secretion. (**A**)—Representative flow cytometry dot plots and quantification of CD19^+^CD24^hi^CD27^+^ Breg cell proportions (upper) and IL-10^+^ cell proportions within the CD19^+^CD24^hi^CD27^+^ gated population (lower) across four treatment groups: Control, SQ22536 (100 μM), TPx (5 μg/mL), and SQ22536 + TPx at 72 h. TPx markedly increased both the Breg proportion (13.0%) and IL-10^+^ cell frequency (4.95%), whereas co-treatment with the adenylyl cyclase inhibitor SQ22536 significantly reduced these effects (10.8% and 3.75%, respectively). (**B**)—IL-10 protein concentrations in culture supernatants across the four treatment groups at 72 h, measured by ELISA; TPx significantly enhanced IL-10 secretion, which was significantly attenuated by SQ22536 co-treatment. Together, these data demonstrate that TPx-induced Breg cell differentiation and IL-10 production are dependent on adenylyl cyclase activity, consistent with engagement of the cAMP/PKA/CREB signaling cascade. Data are presented as mean ± SD. ns, not significant; * *p* < 0.05; ** *p* < 0.01.

Blocking the pathway also impaired TPx-driven Breg function. TPx increased both CD19^+^CD24^hi^CD27^+^ Breg frequency and intracellular IL-10^+^ cells (both *p* < 0.01), and SQ22536 pretreatment significantly attenuated each (*p* < 0.05 and *p* < 0.01, respectively; Figure 6A). Secreted IL-10 mirrored this pattern, with TPx-enhanced supernatant IL-10 reduced by SQ22536 (*p* < 0.01; Figure 6B).

SQ22536 significantly attenuated the TPx-induced increases in intracellular cAMP, PKA expression, CREB phosphorylation, the CD19^+^CD24^hi^CD27^+^ Breg-like population, and IL-10 production. These pharmacological findings support the involvement of adenylyl cyclase activity in the TPx-induced response and demonstrate its association with activation of the cAMP/PKA/CREB axis. However, because SQ22536 may exert pathway-independent effects, these results do not establish the cAMP/PKA/CREB axis as the exclusive mediator of TPx activity.

## 4. Discussion

Cysticercosis is among the neglected tropical diseases prioritized by the WHO for prevention and control, and a central challenge in its management lies in the capacity of cysticerci to establish persistent immune tolerance within the host [36,37]. A growing body of evidence indicates that this immune evasion depends largely on the active modulation of host immune responses by parasite excretory–secretory products [6,38]. Although previous studies have shown that the excretory–secretory antigens of *Cysticercus cellulosae* exhibit potent immunomodulatory activity [6,39], the key effector molecules and their precise mechanisms of action have remained poorly understood. Building on the working hypothesis that specific ESA components mediate this effect, the present study focused on thioredoxin peroxidase (TPx), a protein identified within ESA and demonstrated for the first time that it functions as an immunomodulatory effector: TPx drives B cell differentiation toward the Breg phenotype and promotes IL-10 secretion through the cAMP/PKA/CREB signaling pathway, thereby suppressing CD4^+^ T cell activation and facilitating Treg cell generation.

Breg cells play a critical role in maintaining immune homeostasis by secreting immunosuppressive mediators such as IL-10, IL-35, and TGF-β [40]. Unlike Treg cells, Breg cells lack a lineage-defining transcription factor, and their identification therefore relies on a combination of surface markers and functional characteristics [41]. In humans, CD19^+^CD24^hi^CD27^+^ memory B cells constitute one of the principal Breg subsets enriched for immunoregulatory function [42]. In the present study, TPx significantly promoted the differentiation of this Breg subset and concomitantly upregulated both the transcription and secretion of IL-10. This observation is consistent with the Breg expansion reported across diverse helminth infection models. In patients infected with *Wuchereria bancrofti*, the frequencies of multiple peripheral-blood Breg subsets are markedly elevated [43]. Similarly, in mice infected with *Echinococcus granulosus*, the proportion of splenic CD1d^hi^CD5^+^ Breg cells and serum IL-10 levels are significantly increased, and the parasite’s excretory–secretory products can directly induce B10 cell differentiation in vitro [20,24]. Although these studies differ in parasite species, host, and Breg phenotype, they converge on a shared paradigm: helminths and their secreted products reshape host immunity by inducing Breg expansion and robust IL-10 release. Our findings extend this paradigm by identifying a single, defined molecule that recapitulates it, and we therefore propose that the induction of Breg differentiation and high-level IL-10 secretion represents an important effector mechanism through which TPx mediates host immunomodulation.

A central means by which Breg cells exert immunosuppression is the modulation of T cell responses [44]. Using an in vitro B–T co-culture system, we demonstrated that TPx-primed Breg cells not only directly suppressed CD4^+^ T cell activation and proliferation but also significantly promoted the differentiation of CD4^+^CD25^+^CD127^−^ Treg cells. These results accord with findings from several parasitic infection studies. In a *Babesia* infection model, Breg and Treg cells expand synergistically, and B cell–deficient mice show impaired Treg development and reduced IL-10 levels owing to the loss of Breg function [45]. Studies of *W. bancrofti* infection have likewise confirmed that IL-10–producing Breg subsets suppress Th1/Th17 differentiation and promote Treg conversion, an effect that wanes after anthelmintic treatment [43]. Collectively, these findings suggest that B cells with a regulatory (Breg-like) phenotype may act as upstream regulators that favour the emergence of Treg-like cells and contribute to immune regulation during parasitic infection. On this basis, we propose the following model: TPx first induces and activates Breg cells, which in turn suppress CD4^+^ T cell activation and proliferation and promote Treg generation through paracrine cytokines—notably IL-10—or through direct cell–cell contact, ultimately dampening the host’s protective Th1/Th2 responses. It should be noted, however, that although the GM12878 and Jurkat cell lines used here offer the advantages of proliferative stability and high reproducibility, they differ in biological phenotype from primary cells. Future studies employing co-culture systems based on primary human peripheral-blood cells, as well as in vivo animal infection models, are therefore warranted to further validate these findings.

**Figure 7:**
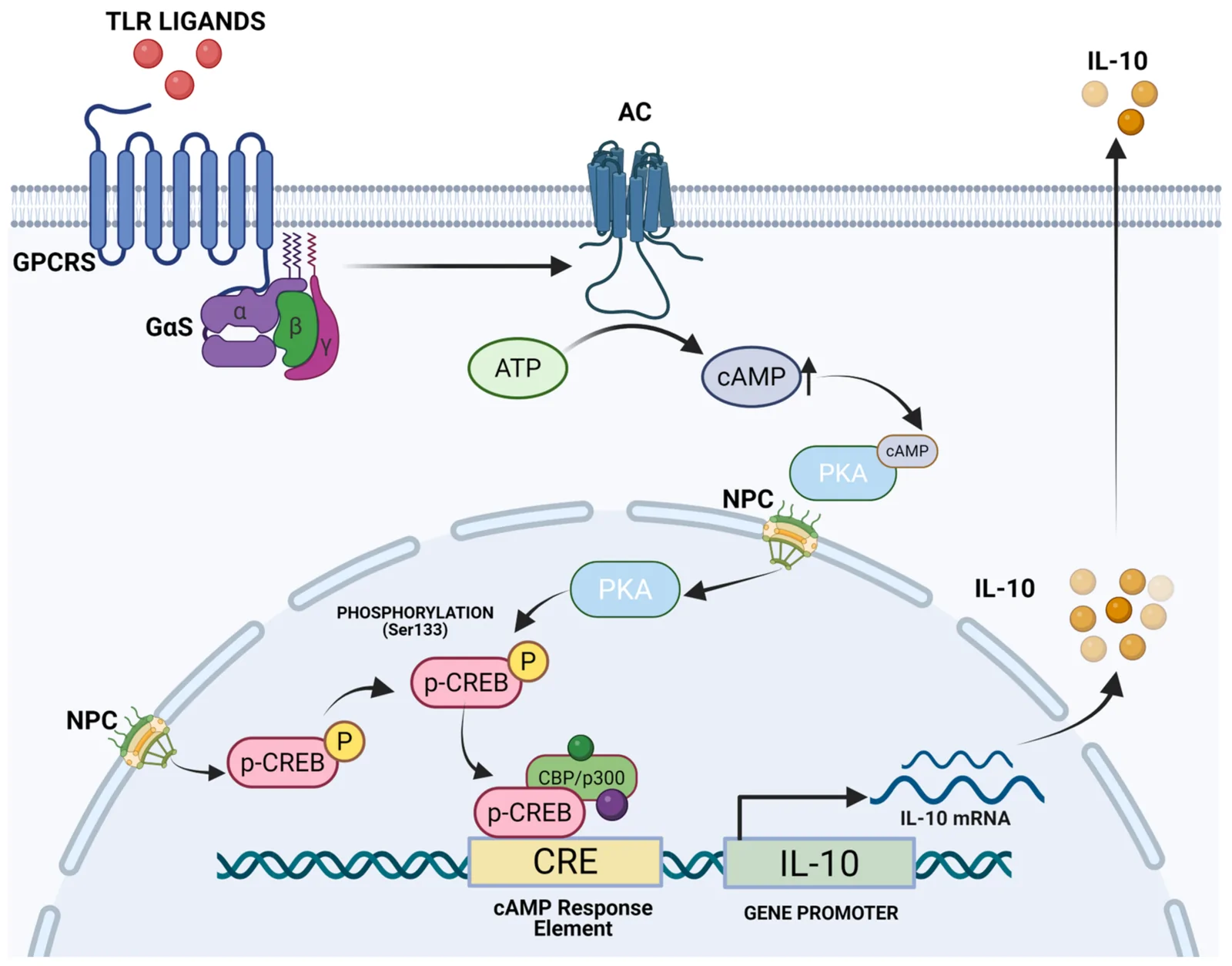
Schematic diagram of the cAMP/PKA/CREB signaling pathway mediating IL-10 transcription. Extracellular stimuli such as Toll-like receptor (TLR) ligands activate G protein-coupled receptors (GPCRs), which signal through stimulatory Gα subunits (Gαs) to activate adenylyl cyclase (AC). AC catalyzes the conversion of ATP to cyclic adenosine monophosphate (cAMP), thereby elevating intracellular cAMP levels. cAMP binds to and activates protein kinase A (PKA), which translocates into the nucleus via the nuclear pore complex (NPC) and phosphorylates the transcription factor CREB at serine 133 (Ser133). Phosphorylated CREB (p-CREB) recruits the transcriptional coactivators CBP/p300, and the resulting complex binds to the cAMP response element (CRE) within the IL-10 gene promoter, initiating IL-10 transcription. The IL-10 mRNA is subsequently translated, and the resulting IL-10 protein is secreted into the extracellular space. This figure was created using BioRender (https://biorender.com, accessed on 4 August 2026).

Guided by transcriptomic evidence, we further established the central role of the cAMP/PKA/CREB signaling axis in TPx-induced Breg differentiation. The IL-10 promoter harbors a conserved cAMP response element (CRE), and CREB is the key transcription factor that binds this element to initiate IL-10 transcription [46,47,48] (Figure 7). This mechanism has been documented in other immune-tolerance settings; for example, prostaglandin E_2_ induces high-level IL-10 expression in macrophages through the EP2/EP4–cAMP–PKA–CREB axis [26,49,50]. In the present study, TPx stimulation significantly upregulated PKA expression and enhanced CREB phosphorylation at Ser133 in B cells, whereas total CREB protein levels remained unchanged. This finding indicates that TPx promotes IL-10 transcription by enhancing CREB phosphorylation and activation rather than by increasing CREB expression. The temporal profile of these signaling events closely paralleled the transcriptomic data and the peak of IL-10 secretion. More importantly, the adenylyl cyclase inhibitor SQ22536 effectively blocked TPx-induced PKA upregulation and CREB phosphorylation and reduced both the Breg proportion and IL-10 secretion to near-control levels. Together, these results firmly establish the cAMP/PKA/CREB pathway as a critical mediator of TPx-induced Breg differentiation and its associated immunosuppressive function.

Viewed in the broadest context, the mechanism elucidated here holds considerable translational value. Given the dual—both protective and pathogenic—role of Breg cells in cysticercosis, a phased therapeutic strategy merits future exploration: preserving the anti-inflammatory function of this pathway during the initial phase of anthelmintic treatment to mitigate treatment-associated inflammatory pathology, and subsequently antagonizing it to break immune tolerance. Beyond parasitic disease, our findings align closely with the “hygiene hypothesis” [51,52,53], as epidemiological evidence has revealed a significant inverse correlation between helminth infection and the incidence of conditions such as asthma, inflammatory bowel disease, and multiple sclerosis [54,55,56]. Compared with live “helminth therapy,” which faces considerable ethical and safety challenges [57,58], the development of individual parasite-derived molecules as substitutes for live infection offers substantial advantages in defined composition, controllable dosage, and a favorable safety profile [59]. As a well-characterized functional molecule with a defined dependence on the cAMP/PKA/CREB axis for inducing immune tolerance, TPx not only provides a theoretical basis for developing novel immunomodulatory biologics but also offers new perspectives for designing targeted small-molecule therapeutics for allergic and autoimmune diseases.

Several limitations should be acknowledged. First, GM12878 is an EBV-transformed lymphoblastoid cell line whose constitutive viral signaling may influence basal IL-10 expression and TPx responsiveness. Moreover, because TPx was evaluated only in CpG-preactivated cells, its independent effect cannot be distinguished from modulation of a CpG-induced response. Although recombinant TPx contained less than 0.01 EU/mL endotoxin, the absence of heat-inactivated TPx, catalytically inactive TPx, and mock-purification controls prevents complete exclusion of contaminant-, conformation-, or enzymatic activity-related effects. The reduced response at 50 μg/mL also requires further viability and aggregation assessments. Second, SQ22536 inhibition supports an adenylyl cyclase-sensitive response associated with cAMP/PKA/CREB activation but does not establish pathway specificity. Because CREB-specific knockdown or inhibition and cAMP-analogue rescue were not performed, other cAMP effectors cannot be excluded. CREB siRNA or genetic disruption, CREB-specific inhibition, and rescue with 8-Br-cAMP or db-cAMP will be required to establish CREB dependence [60]. Finally, Jurkat cells and PMA/ionomycin do not reproduce physiological CD4^+^ T-cell activation; FSC-A is not a direct proliferation measure, and CD4^+^CD25^+^CD127^−^ expression alone does not establish bona fide Treg differentiation. Furthermore, because IL-10 receptor blockade, TGF-β or IL-35 neutralization, and transwell experiments were not performed, the contributions of individual cytokines and contact-dependent mechanisms remain unresolved. Future studies using primary B and CD4^+^ T cells, cytokine-specific blockade, functional assays, and in vivo validation are therefore required. In addition, in vivo evaluation of the therapeutic efficacy of TPx in animal models of autoimmune or allergic disease represents a promising direction that warrants further investigation.

## 5. Conclusions

In this exploratory cell-line model, *C. cellulosae* TPx promoted an IL-10-producing CD19^+^CD24^hi^CD27^+^ Breg-like phenotype in CpG-preactivated GM12878 cells and altered activation-associated features of co-cultured Jurkat cells. The response was accompanied by increased cAMP, PKA Cα abundance, and CREB Ser133 phosphorylation and was attenuated by SQ22536, supporting dependence on adenylyl cyclase activity and an association with the cAMP/PKA/CREB axis. These findings identify TPx as a candidate mediator of helminth-driven B-cell immunoregulation; however, studies in primary B cells using pathway-specific rescue, genetic perturbation, mediator blockade, and in vivo models are needed to establish the mechanism and physiological relevance.

## Data Availability

The original contributions presented in this study are included in the article/Supplementary Materials. Further inquiries can be directed to the corresponding author.

## Supplementary Materials

The following supporting information can be downloaded at https://www.mdpi.com/article/10.3390/cells15161429/s1: Table S1: Primer sequences used for RT-qPCR analysis; Table S2: Number of differentially expressed genes, Figure S1. SDS-PAGE analysis of the TPx protein.
