# Supplementary material for: The TPx Protein of Cysticercus cellulosae Promotes an IL-10–Producing Regulatory B Cell Phenotype in Human B Lymphoblastoid Cells via the cAMP/CREB Pathway
Source: Cells. 2026 Aug 8;15(16):1429. doi: 10.3390/cells15161429 (PMC13511132; doi:10.3390/cells15161429)

Supplementary data

Table S1. Primer sequences used for RT-qPCR analysis.

Gene	Primer	Sequence (5′-3′)
IL-10	Forward	CAAGCCTTGTCTGAGATGATCCA
	Reverse	TGTCAAACTCACTCATGGCTTTG
IL-12A	Forward	AGACCATGAATGCAAAGCTTCTG
	Reverse	TCAATAGTCACTGCCCGAATTCT
TGFβ	Forward	TACAGCAACAATTCCTGGCGATA
	Reverse	CAGTGTGTTATCCCTGCTGTCAC
GZMB	Forward	TGACAGTGCAGGAAGATCGAAAG
	Reverse	TCCAGTGTACAAAGCTTGAGACT
GAPDH	Forward	GGAGTCCACTGGCGTCTTCA
	Reverse	GTCATGAGTCCTTCCACGATACC

Table S2. Number of differentially expressed genes.

Compared groups	Regulated type	p-value<0.05&|log2FC|>0.58
**TPx_48h-vs-Control_48h**	Forward	86
	Reverse	80
**TPx_72h-vs-Control_72h**	Forward	69
	Reverse	55

Figure S1. SDS-PAGE analysis of the TPx protein.

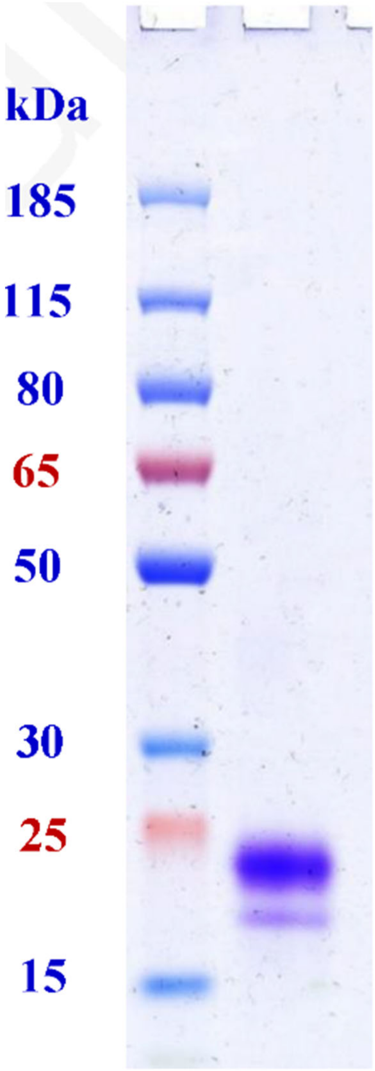

Supplement: Supplementary file 1 [file cells-15-01429-s001.zip › cells-4447468-supplementary.pdf]
